# Multidimensional daily diary of fatigue–fibromyalgia–17 items (MDF-fibro-17). part 1: development and content validity

**DOI:** 10.1186/s12891-017-1544-y

**Published:** 2017-05-16

**Authors:** S. Morris, Y. Li, J.A.M. Smith, S. Dube’, C. Burbridge, T. Symonds

**Affiliations:** 1 Former employees, current consultants of Theravance Biopharma US, Inc, 901 Gateway Boulevard, South San Francisco, CA 94080 USA; 2 Former employees of Theravance Biopharma US, Inc, 901 Gateway Boulevard, South San Francisco, CA 94080 USA; 3 Consulting Associate Professor Department of Psychiatry, Behavioral Medicine Stanford University School of Medicine, Stanford, CA 94305 USA; 4 Adjunct Professor of Psychiatry University of Pittsburgh School of Medicine, Pittsburgh, PA 15260 USA; 5 Clinical Outcomes Solutions, Unit 68 Basepoint, Shearway Business Park, Shearway Road, Folkestone, Kent, UK

**Keywords:** Fibromyalgia, Fatigue, Diary, Patient reported outcome (PRO), Qualitative, Conceptual model, MDF-Fibro-17

## Abstract

**Background:**

Fibromyalgia (FM), a disorder characterized by chronic widespread pain and tenderness, affects greater than five million individuals in the United States alone. Patients experience multiple symptoms in addition to pain, and among them, fatigue is one of the most bothersome and disabling. There is a growing body of literature suggesting that fatigue is a multidimensional concept. Currently, to our knowledge, no multidimensional Patient Reported Outcome (PRO) measure of FM-related fatigue meets Food and Drug Administration (FDA) requirements to support a product label claim. Therefore, the objective of this research was to evaluate qualitative and quantitative data previously gathered to inform the development of a comprehensive, multidimensional, PRO measure to assess FM-related fatigue in FM clinical trials.

**Methods:**

Existing qualitative and quantitative data from three previously conducted studies in patients with FM were reviewed to inform the initial development of a multidimensional PRO measure of FM-related fatigue: 1) a concept elicitation study involving in-depth, open-ended interviews with patients with FM in the United States (US) (*N* = 20), Germany (*N* = 10), and France (*N* = 10); 2) a cognitive debriefing and pilot study of a preliminary pool of 23 items (*N* = 20 US patients with FM); and 3) a methodology study that explored initial psychometrics of the item pool (*N* = 145 US patients with FM).

**Results:**

Five domains were identified that intend to capture the broad experience of FM-related fatigue reported in the qualitative research: the Global Fatigue Experience, Cognitive Fatigue, Physical Fatigue, Motivation, and Impact on Function. Seventeen of the original pool of 23 items were selected to best capture these five dimensions. These 17 items formed the basis of a newly developed multidimensional PRO measure to assess FM-related fatigue in clinical trials: the Multidimensional Daily Diary of Fatigue-Fibromyalgia-17 (MDF-Fibro-17).

**Conclusion:**

Qualitative analysis, and preliminary quantitative item level data, confirmed that FM-related fatigue is multidimensional and provided strong support for the content validity of the MDF-Fibro-17. The next stage was to quantitatively evaluate the measure to confirm the factor structure, psychometric properties, sensitivity to change, and meaningful change. This has been conducted and is being reported separately.

## Background

Fibromyalgia (FM) is a disorder characterized by chronic widespread pain and tenderness that predominantly affects women. It is estimated to affect approximately five million individuals, or about two to three percent of the general population in the United States (US) alone [1–4]. Worldwide estimates vary, ranging between 0.5 – 10% of the population [5, 6]. In addition to widespread pain, patients with FM often experience other symptoms, such as fatigue, impaired sleep, negative mood, cognition limitations, and physical functioning limitations. These difficulties are associated with a reduced overall health-related quality of life (HRQoL), as well as lowered productivity, unemployment, and disability [7–9]. Fibromyalgia is a complex condition associated with multiple symptoms that are inter-related. This is reflected in qualitative work in which individuals describe their experiences of living with a complex array of symptoms on a daily basis [10, 11]. Beyond pain, fatigue is commonly identified as one of the most bothersome and disabling symptoms in FM with more than 80% of patients describing fatigue as “disruptive or extremely disruptive” to their HRQoL [1, 2, 12–15].

There is evidence that, although related, fatigue and pain are distinct both from each other and from other FM-related symptoms [10, 14–18]. This evidence is further bolstered by study results that indicate a specific effect of treatments upon fatigue compared to other FM symptoms, such as pain and sleep in clinical trials, suggesting that fatigue is not solely driven by changes in pain, or other symptoms [11]. Treatments that improve pain or sleep do not necessarily improve fatigue, thus, a comprehensive FM clinical trial should assess fatigue as well as pain or other symptoms [19–23].

In analyses of clinical trial data, fatigue has been shown to be a key driver of how patients’ assess their overall health status [12, 13]. The importance of fatigue in FM is also recognized by experts in the field; 94% of participants at the Outcome Measures in Rheumatology (OMERACT) Seven workshop, comprised of clinicians, researchers, regulatory and industry representatives, agreed that fatigue was an essential domain to measure in FM clinical trials [17]. Yet, fatigue is not consistently included as an endpoint in FM clinical trials, and the primary endpoint in the pivotal trials of agents recently approved for FM has been reduction in pain and global experience, not fatigue [18]. The absence of FM-related fatigue claims likely reflects both the lack of conclusive evidence of a fatigue treatment effect among the currently available treatment options [16, 24–27], and an absence of accepted measurement tools. Although several measures of fatigue have been included in clinical studies (such as the Multidimensional Fatigue Inventory) [28], at this time we are not aware of any existing measure that has been established as “fit for purpose” for evaluating fatigue in FM.

The FDA guidance for the development of a patient reported outcome (PRO) measure to support a label claim, outlines the need for in-depth qualitative work with the target population to inform the development of, and demonstrate the content validity of a PRO tool [29]. Such in-depth qualitative work has already been conducted in patients with FM, which confirmed the importance of fatigue in FM [14]. Although this work was originally used to support the development of a brief, unidimensional measure designed to capture only the core global symptoms of FM-related fatigue - the Daily Diary of Fatigue Symptoms (DFS-Fibro), multidimensional aspects of FM-related fatigue were clearly identified [6]. A pool of 23 items was developed to reflect the multidimensional aspects of fatigue, which were initially grouped into proposed domains capturing the severity (overall fatigue, characterizing fatigue, physical body fatigue, and motivation) and impact (daily activity limitations and cognitive limitations) of fatigue [6]. These items were cognitively debriefed and included within a pilot study to gather empirical data on the items.

There is increasing recognition and acceptance of fatigue as a multidimensional construct, and an interest in examining whether investigational treatments may impact specific dimensions of fatigue (ie, cognitive fatigue, physical fatigue). Therefore, the current authors wanted to explore the development of a measure that would capture the complexity of FM-related fatigue, while also allowing assessment of different dimensions of fatigue. In the current study, the authors utilized the existing qualitative and quantitative data on the multidimensional item pool, as a preliminary stage in the development of such a multidimensional measure.

### Objective

The study objective was to further evaluate qualitative and quantitative data previously gathered in order to gain a full understanding of the patient experience of FM-related fatigue, and to develop a multidimensional PRO measure of FM-related fatigue for use in clinical studies. The study aims were to a) confirm the multidimensional components of FM-related fatigue, b) explore the understanding, interpretation, and relevance of the 23 item pool, derived from earlier elicitation interviews using the patients’ language to describe the complex experience of FM-related fatigue, c) explore item redundancy and performance in order to optimize scale sensitivity and concept saturation, and d) to propose a list of items and potential themes or domains, that would be suitable for inclusion within a multidimensional measure of FM-related fatigue.

## Methods

### Previous qualitative studies

The present research was based on data gathered over the three studies that had previously been conducted for the development of an earlier measure of fatigue in FM. These studies included a concept elicitation study, a cognitive debriefing study, and a cross-sectional methodology study. The outcomes from these studies included a 23-item pool describing the experience of fibromyalgia-fatigue, and a five-item global fatigue measure. In these three studies, all participants were adults who met the American College of Rheumatology criteria for FM [2], with no significant co-morbidities that could confound reporting of FM. The concept elicitation study was conducted with 40 individuals with FM in the US (*n* = 20), Germany (*n* = 10) and France (*n* = 10). Key themes and concepts were identified using qualitative analysis methods based upon grounded theory. The cognitive debriefing and pilot testing of the 23-item pool was conducted in an additional 20 individuals with FM in the US only. The cross-sectional methodology study included a total of 145 patients with FM in the US, who ranked their symptom severity using the 23 items administered on an electronic daily diary for two weeks. The data was then used to evaluate the psychometric properties of the 23 item-pool. Further details of the methodology, original analyses, and results of these three studies involved have been published elsewhere [6, 14].

### The current study

In the current study, the original concepts identified in the concept elicitation interviews, the feedback that was obtained on items from the cognitive debriefing, and the item level quantitative data from the original studies were reviewed to inform the development of a multidimensional tool.

The qualitative re-analysis involved a review of the original transcripts and analytical coding to confirm that the patient data supported the importance of all aspects of fatigue, and that the 23 items reflected the qualitative findings and language used by the patients. The qualitative and quantitative data on each item was also reviewed to confirm patient understanding and item performance as expected. Based on this work, a 17-item, multidimensional measure was proposed. A confirmatory factor analysis was then conducted to explore the fit of items to the multidimensional structure proposed in the initial conceptual model. This re-analysis was conducted by SM and YL, and findings were reviewed with those involved in the original analysis (CB, TS) and other experts in the area (JS, SD).

## Results

The review that was conducted in the current study of the original qualitative transcripts and qualitative data analysis confirmed the relevance of each of the key elements of fatigue in FM to the patients and supported the appropriateness of a multidimensional assessment of FM-related fatigue to capture the full complexity of this experience. All of the key concepts were clearly identified within the initial concept elicitation interviews and items within each of the key concepts were reported as being relevant and well understood by patients upon cognitive debriefing.

Five domains of FM-related fatigue were confirmed upon review of the qualitative data: Global Fatigue Experience, Cognitive Fatigue, Physical Fatigue, Motivation, and Impact on Function. These domains were based upon the key themes identified from the qualitative analysis, and confirmed through item analysis and confirmatory factor analysis. Together, these five domains captured the key elements of FM-related fatigue that were being reported by patients, across all three countries, during the concept elicitation interviews. Results of the qualitative and quantitative analyses, confirming this multidimensional structure, are discussed below.

### Global fatigue experience

A single domain of “global fatigue experience” was identified to capture global and general descriptors of FM-related fatigue that appeared to transcend the other, more specific, domains. Patients used a variety of general terms to describe their fatigue. For example, patients would describe their fatigue in the following ways:

*“When tiredness happens, there’s no relief. It’s like an overwhelming, overarching, penetrating, consuming tiredness. There’s nothing you can do about it.” (003-005, US, Female)* [10]

*“The absolute tiredness just, for no reason that can strike at any time. It’s like you’re totally exhausted mentally and physically.” (003-004, US, Female)* [10]

*“I would feel tired, or exhausted, or just run down, those kind of terms, which is all connected.” (001-001, US, Female)* [10]

*“And now to do the simplest little thing, I can be where I’m just totally worn out. Like I’ve been doing something all day long very strenuous.” (003-004, US, Female)* [10]

No single term was identified that adequately captured the global experience of FM-related fatigue in the same way for all. Therefore, a domain containing multiple items using global terms was felt to be an important element of a comprehensive PRO measure to ensure that it captured the global experience of fatigue using patient derived language. All patients reported experiencing fatigue as part of their FM.

### Cognitive fatigue

Patients asserted that FM-related fatigue affected them both physically and mentally. Cognitive limitations were described as an integral part of FM-related fatigue. In the concept elicitation interviews, patients often differentiated between FM-related fatigue and normal tiredness by the impact of the former on concentration, thinking clearly and remembering, and used this to explain the severity of their fatigue.

*“It can be difficult, in the sense that the fatigue makes it hard to concentrate. That’s for sure.” (004-007, France, Female)* [10]

*“It’s like if something happens while I’m fatigued, I have brain fog. I forget about it. But if I do something when I’m clearheaded, then I’ll remember that as a memory.” (006-004, US, Female)* [10]

In the concept elicitation interviews, 21 patients (52.5%) talked about a mental or cognitive component to their fatigue.

### Physical fatigue

The physical aspect of FM-related fatigue was also described as an important part of FM-related fatigue throughout the interviews. In the concept elicitation interviews, 16 patients (40%) talked about their body feeling heavy, nine patients (22.5%) talked about their muscles feeling weak, and 32 patients (80%) described experiencing tiredness in specific areas of their body.:

*“My body can feel very heavy. Like I have to drag to move, because I’m so tired.” (002-002, US, Female)* [10]

*“Interviewer: Could you describe the feeling of fatigue?” Patient response: “Just tired and heavy and draggy and slow.” (001-005, US, Female)* [10]

*“I would think about the energy it takes, and I would feel – I just feel like weighted down. That’s what the fatigue feels like. It’s almost a weak feeling, or a heavy feeling.” (006-005, US, Female)* [10]

### Motivation

Patients consistently described motivation as a direct manifestation, and integral part, of FM-related fatigue, and appeared to link this to severity of fatigue on a daily basis. Difficulties with motivation were described as difficulty “getting going,” or as an increase in effort to conduct activities. Although such comments may reflect a lack of motivation, or an underlying mood disorder such as depression, it is important to note that patients with comorbid depression were excluded from the study to avoid any confounding of symptoms in the patient experience.

*“But the other thing is, the energy, to me, also is connected to motivation…it decreases, at least for me, the motivation to do things. And that’s what I find very frustrating.” (001-001, US, Female)* [10]

*“I can’t do everything because I’m extremely tired.” (004-002, France, Female)* [10]

*“Yes, well, the lack of enthusiasm which I have, that I have to overcome to motivate myself into doing something.” (005-004, Germany, Female)* [10]

In the concept elicitation interviews, 19 patients (48%) across all three countries discussed the impact of fatigue using general terms such as describing an “increased effort to do things,” “forcing oneself to do things,” and having trouble “getting motivated to do things.” More specifically, for 16 patients (40%), the concept of motivation was also discussed in relation to getting out of bed in the morning or having to “get going” in the morning.

### Impact on function

A clear message from the interviews was that patients with FM often communicate their experience of FM by describing how fatigue negatively impacts their functional capacity, including their ability to conduct daily activities. Patients often used general statements referring to “things” as an overarching evaluation of impact, rather than specific activities. When specific activities were mentioned during a probed discussion, these were numerous, with 17 categories of activity identified, and no individual activity was reported by more than 7.5% of the overall population across the three countries. Therefore, an Impact of Function domain, using “doing things” as a general descriptor, was felt to be important in a comprehensive measure of FM-related fatigue and included in the final conceptual model.

*“I mean, you just have no energy to get up and go any place. Like to work or any activities or to make dinner or just do anything.” (001-002, US, Male)* [10]

*“It’s a slowing down in being able to do things. Like where it would normally take me a minute, two minutes to wash a plate…the first one may start out that way, but as I start going through the dishes, I start to slow down.” (002-002, US, Female)* [10]

Overall, 27 patients (67.5%) in the concept elicitation interviews spontaneously talked about their fatigue/tiredness affecting general things they wanted to do, and 24 patients (60%) talked about their fatigue/tiredness specifically affecting their daily activities.

### The five domain, multidimensional daily diary of fatigue in fibromyalgia (MDF-fibro-17)

The data from the cognitive debriefing of the original pool of 23 items, capturing all elements of fatigue that emerged from the qualitative work, was then re-evaluated in light of this multidimensional framework. The patient feedback confirmed that most items were clearly understood and considered to be relevant, and overall supported using 17 of the original pool of 23 items to capture these five dimensions of FM-related fatigue. The six items that were flagged for exclusion from the item pool, along with the rationale for their exclusion, are provided in Table 1. The decision to remove the items was based on the qualitative and quantitative data. Findings from the cognitive debriefing revealed a lack of conceptual clarity, and a tendency to over-represent severe symptoms. The item level analysis data from the cross-sectional validation study confirmed the poor performance of these items that had been flagged for removal. One item (energy) had low correlations with all other items (0.177 to 0.339), and the others had high correlations (all above 0.84) with other items within their target domain, indicating potential item redundancy. These items also showed a tendency towards a “floor” effect. Taken together, these findings lead to the removal of the six weakest items from the item pool.

**Table 1:** Rationale for deleted items

Item	Original domain (Humphrey et al) [14]	Rationale for removal
How much of the day were you unable to do things due to your fatigue?	Overall fatigue	• The term “unable” appeared to target more severe fatigue only, based on qualitative feedback and a tendency towards a stronger floor effect than other items within domain. • Patients reported some confusion about how to answer, for e.g. often they could still do things, even if difficult. • Redundant item, highly correlated with other items (all above 0.86 with other items from the global fatigue experience domain). This was the least clearly understood item.
How much energy did you have today?	Characterizing fatigue	• Performed poor psychometrically, low correlations with other items (0.177 to 0.339). Most likely due to the fact that it is the only positively stated item. • Interviews indicate “energy” may mean different things to different people (e.g. physical versus mental).
How much did tiredness stop you from doing things today?	Activity limitations	• As with “unable”, the term “stop you” appeared to target more severe symptoms only, based on qualitative feedback and a tendency towards a stronger floor effect than other items within domain. All correlations above 0.944 with other items in the impact on function domain.
How tired did you feel when you woke up in the morning?	Characterizing fatigue	• Intention of question was to capture “unrelenting fatigue not improved with sleep”, however not clearly interpreted as such. • Quantitative data suggested that question conceptually overlapped with separate concept of sleep quality rather than fatigue.
How weak did you feel today?	Characterizing fatigue	• Redundant with question about “how weak did your muscles feel today?” (correlation 0.967) This was the least clearly understood question. • Quantitative data suggested that this item was somewhat conceptually confusing.
How much of the day did you feel overwhelmed by tiredness?	Characterizing fatigue	• Interpreted as targeting more severe fatigue based on the qualitative feedback and a tendency towards a stronger floor effect than other items within domain. • Conceptually unclear. Some patients felt that the question was asking about an emotional component of fatigue.

Based on these results, a 17-item multidimensional measure of FM-related fatigue has been developed, capturing five domains: Global Fatigue Experience (four items); Cognitive Fatigue (four items); Physical Fatigue (three items); Motivation (three items); and Impact on Function (three items).

**Fig. 1:**
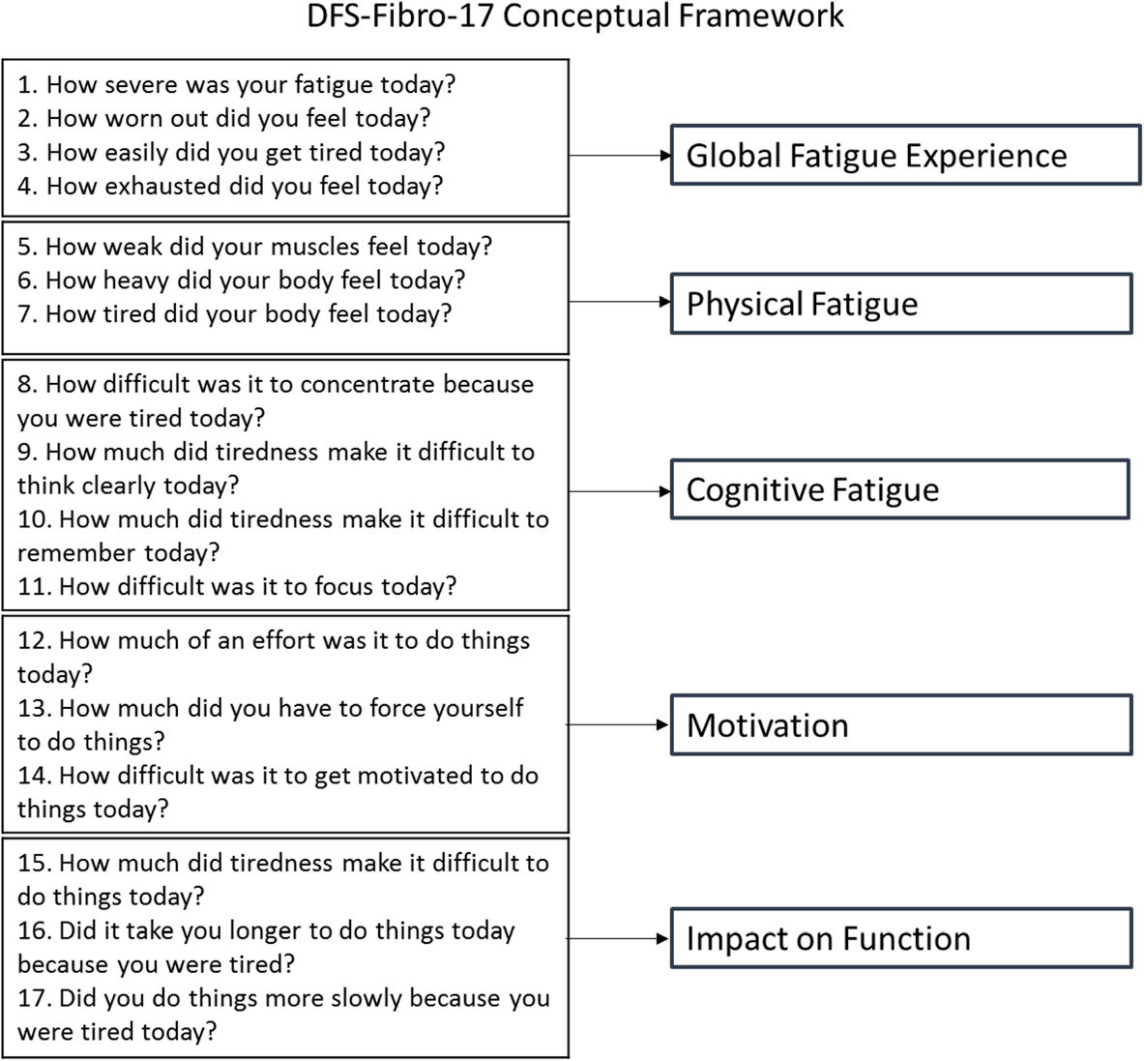
DFS-Fibro-17 conceptual framework

The revised multidimensional conceptual model of FM-related fatigue, and framework for the MDF-Fibro-17 is presented in Fig. 1. Amongst these 17 items, there were no floor or ceiling responses observed (1.4 – 11.3% selecting zero, and 2.1 – 9.5% selecting ten), and a relatively even spread of responses across the 0 to 10 scale (mean scores ranged from 4.2 to 6.3, with a minimum of zero and a maximum of ten on all items). Inter-item correlations were high (all above 0.720), and when evaluating correlations between items within a domain, all were above 0.889. Many inter-item correlations were above 0.9, suggesting potential item redundancy, however the qualitative data confirmed the relevance of the multidimensional aspects of FM-fatigue from the patient perspective. Therefore, items that were otherwise supported through the qualitative work were retained despite the high inter-item correlation to maintain the comprehensive nature of the proposed measure. An initial confirmatory factor analysis was conducted using the dataset obtained from the cross-sectional validation study confirmed that the 17-item model performed better than all 23 items. The 17 item, multi-dimensional model resulted in an appropriate fit on all fit measures, including comparative fit index (CFI) = 0.96 and a root mean square error of approximation 0.08. These results are shown in Table 2.

**Table 2:** Initial CFAs of Item-level results

	×^2^	CFI	NNFI	RMSEA (90% CI)	SRMR
Initial Validation Study (*n* = 138)					
MDF-Fibro k = 17, Five-Factor Model	198.17 (df = 109)	0.96	0.95	0.077 (0.060 – 0.094)	0.016

*CFI* comparative fit index, *CI* confidence interval, *NNFI* non-normed fit index, *RMSEA* root mean square error of approximation, *SRMR* standardized root mean square residual

## Discussion

A 17-item, multidimensional PRO measure of FM-related fatigue has been developed based upon this review of the existing qualitative work and 23-item pool. The measure was developed in response to the growing evidence supporting the multidimensional nature of FM-related fatigue, and the absence of a “fit for purpose” multi-dimensional PRO measure of FM-related fatigue. The authors utilized a rich source of in-depth, qualitative data that had been gathered as part of original research involving the direct input from the patient group, in accordance with the FDA PRO Guidelines for the development of PRO measures [14, 29] and preliminary item level data that had been obtained from a cross-sectional validation study involving the administration of the original 23-item pool. This data was used to support qualitative and quantitative exploration of the pool of items, and the preliminary validation of multifactorial aspects of fatigue in FM.

The review of the original qualitative data supports the multi-dimensional nature of FM-related fatigue and confirms the relevance and appropriateness of each of the key elements in the model: Global Fatigue Experience, Physical Fatigue, Cognitive Fatigue, Motivation, And Impact on Function. This data provides strong evidence of content validity of the MDF-Fibro-17.

In relation to the previous theoretical grouping of the 23 item pool initially published [6], two of the original domains (“overall fatigue” and “characterizing fatigue”) were combined to form the Global Fatigue Experience domain in the current conceptual model. The authors felt that, based on the qualitative data, this combination of items better represented the general descriptors of fatigue that patients often used to describe a global sense of fatigue. Otherwise, the domains reflect those originally proposed in the initial research, with six items from the initial pool being removed as described.

This structure of the MDF-Fibro-17 is assumed to be an accurate representation of the key concepts in FM-related fatigue. This assumption is based upon the categorization of concepts developed through a review of the grounded theory analysis of the qualitative work, with additional support from the cognitive debriefing work, which indicated that the items within the measure were relevant and understandable. The item level data and initial confirmatory factor analysis, conducted using data from the previous cross-sectional validation study, also supported the content validity of the proposed MDF-Fibro-17. The psychometric evaluation of the tool is limited by the non-interventional research design and lack of longitudinal data. However, further evaluation of the factor structure, reliability, construct validity, and sensitivity to change of the multidimensional measure has been conducted, and results are pending publication. The ability to explore any subgroup differences in FM-related fatigue is limited in this work because of the sample size. As was noted within the original publication of the qualitative work [14], it would also be useful to explore gender or cultural differences in the experience and reporting of FM-related fatigue further.

To date, studies investigating fibromyalgia-related fatigue have used generic measures of fatigue. Such measures have not been developed specifically for fibromyalgia, however our findings are consistent with the findings of other research conducted in the area. The multidimensional structure of the MDF-Fibro-17, developed in the current study based upon work directly involving patients with FM, is very similar to the domains captured within the Multidimensional Fatigue Inventory (MFI; Smets et al, 1995), a generic measure of fatigue [28]. Arnold et al. (2011) utilized the MFI to demonstrate the impact of duloxetine treatment on fatigue in patients with FM, and showed an observed change on all domains on the measure [12]. This supports the use of a multidimensional measure of fatigue, which captures both the physical and mental components of fatigue, and demonstrates the usefulness of such a measure in providing a richer, more in-depth assessment of the experience of fatigue in FM. Future work should investigate whether the MDF-Fibro-17, developed based on input from individuals with fibromyalgia, provides more in-depth or sensitive results than general measures of fatigue.

## Conclusions

A 17-item multidimensional PRO measure assessing FM-related fatigue (MDF-Fibro-17) for use in clinical trials has been developed in accordance with FDA PRO regulatory guidance. The MDF-Fibro-17 consists of five domains (Global Fatigue Experience, Cognitive Fatigue, Physical Fatigue, Motivation, and Impact on Function), which capture all aspects of the broad patient experience of FM-related fatigue. The current re-evaluation of existing qualitative and quantitative data suggests that the MDF-Fibro-17 has strong content validity and captures the breadth of the experience of FM-related fatigue for the patient. The next stage is to confirm the factor structure, ensure sound psychometric properties, evaluate sensitivity to change, and explore meaningful change on this measure in a clinical trial of patients with FM. The tool has been incorporated into a Phase 2 clinical trial in patients with FM to explore and confirm these measurement properties. These analyses have been conducted, and results are to be published

## Abbreviations

CFI: Comparative fit index
DFS-Fibro: Daily diary of fatigue symptoms - fibromyalgia
FDA: Food and drug administration
FM: Fibromyalgia
HRQoL: Health-related quality of life
MDF-Fibro-17: Multidimensional daily diary of fatigue-fibromyalgia-17 items
OMERACT: Outcome measures in rheumatology
PRO: Patient reported outcome
RMSEA: Root mean square error of approximation
SEALD: Study endpoints and label development
US: United States
